# A 3-nitro triazole as a hypoxic cell sensitizer.

**DOI:** 10.1038/bjc.1983.18

**Published:** 1983-01

**Authors:** M. B. Astor, J. C. Parham, E. J. Hall, M. A. Templeton, B. Hartog


*Br. J. Cancer* (1983), **47**, 155–157

### Short communication

### A 3-Nitro triazole as a hypoxic cell sensitizer

M.B. Astor*, J.C. Parham†, E.J. Hall*, M.A. Templeton† & B. Hartog*

**Radiological Research Laboratory, College of Physicians & Surgeons of Columbia University, New York, N.Y. 10032, †Walker Laboratory, Sloan-Kettering Institute, Rye, New York 10580, U.S.A.*

Many combinations of ring structures and sensitizing groups have been synthesized and tested for potential use as radiotherapy adjuvants to overcome the resistance of hypoxic tumour cells to ionizing radition. Among the drugs tested have been nitroxyl compounds Cooke *et al.* (1976), nitrofurans Chapman *et al.* (1973), nitropyrroles Raleigh *et al.* (1978), nitroimidazoles Asquith *et al.* (1974), non-nitro compounds Wardman *et al.* (1982), and indoles Infante *et al.* (1980). Of the compounds examined, misonidazole (MISO) and desmethylmisonidazole, have entered clinical trials, but optimal doses of drug with each radiation dose fraction are not possible due to a cumulative neurotoxicity.

MISO mimics oxygen and functions as a radiosensitizer because of its electron affinity; this is the so-called "rapid" component of biological sensitization Adams, (1982). A prolonged incubation of MISO under hypoxia leads to the depletion of cellular thiols and results in an additional component of radiosensitization (the biological "slow" component) as well as a sensitization to some chemotherapy agents Stratford *et al.* (1980). The extent to which MISO and other similar compounds produce the preincubation effects correlates well with the amount of cell killing produced, i.e. the chemosensitization from prolonged hypoxic incubation is linked inexorably with cytotoxicity.

In the search for compounds superior to MISO, it might prove fruitful to consider separately the requirements for radiosensitization and chemosensitization. In the case of chemosensitization a compound is needed that reacts rapidly to deplete cellular thiols. In this case hypoxic cell cytotoxicity is probably unavoidable, although we do not know whether there is either a direct link, or even a one-to-one correlation, between cytotoxicity in the petri dish and the troublesome dose-limiting neurotoxicity in the human. For radiosensitization alone, a compound showing minimal cytotoxicity may be advantageous which could then be used at much higher concentrations. This is the approach used in the development of the triazole (1-methyl-3-nitro-1,2,4 triazole 3-NTR), tested in this report.

Chinese hamster V79 cells grown in Ham's F10 supplemented with 10% foetal bovine serum, antibiotics and L-glutamine were used in this study. The procedure for the treatment of cells has been described previously. Briefly, log phase cells grown in Corning 150 $cm^2$ tissue culture flasks were trypsinized and resuspended in complete growth medium. Aerated and hypoxic cells at a concentration of $2 \times 10^5$ cells $ml^{-1}$ were treated in glass spinner vessels based on the design of Chapman *et al.*, 1974. Hypoxia was induced by degassing with high purity $N_2$ for a period of 1 h, followed by addition of degassed drug to obtain the desired final drug and cell concentration. The cells were then incubated at 37.5°C for 1 h with drug prior to irradiation or samples removed at desired intervals for cytotoxicity determinations. Aliquots were plated into flasks with fresh medium, incubated for 7 days and fixed and stained.

The X-ray source was a Sieman's Stabilipan; 300 k Vp, 12 mA, 0.2 mm Cu; based on measurement with a Victoreen ionization chamber, the dose-rate at the treatment distance of 25 cm was calculated to be 6.3 Gy $min^{-1}$.

Data in Figure 1 are from a representative experiment in which hypoxic or aerated V79 cells were treated with drug at 37.5°C for a period of 1 h and during the immediately following exposure to graded doses of X-rays. Figure 2 comprises pooled data from several experiments (including those in Figure 1) expressing the enhancement ratio (ER) as a function of drug concentration. ER is defined as the ratio of doses for cells treated under hypoxic conditions without drug to hypoxic cells treated with drug to obtain an equal biological effect. Survival data for hypoxic or aerated V79 cells treated at 37.5°C with 3-NTR are shown in Figure 3.

The data show that 3-NTR is a very efficient radiosensitizer, specific for hypoxic cells, needing approximately one-half the concentration of MISO to obtain an equal ER. Furthermore, 3-NTR at the

Received 23 September 1982, accepted 7 October 1982.

0007-0920/83/010155–03 $01.00

© The Macmillan Press Ltd., 1983

**Figure 1** Representative radiation survival data for hypoxic and aerated V79 cells treated with various concentrations of 3-NTR for a period of 1 h at 37.5°C prior to exposure to graded doses of X-rays. The curves were fitted to the data by eye.

**Figure 2** The enhancement ratio (ER) as a function of the concentration for the drugs 3-NTR and MISO. The results are data pooled from several experiments.

**Figure 3** Cell survival data for hypoxic and aerated V79 Chinese hamster cells incubated at 37.5°C in the presence of 3-NTR or MISO.

highest dose tested, exhibited no significant toxicity towards either aerated or hypoxic cells.

This communication presents new data in the search for an alternative compound for use as an adjuvant to radiotherapy. The sensitizing efficiency of aromatic nitro compounds is directly correlated with their electron affinity. These properties are commonly increased by introducing electron withdrawing substituents on the ring. A second method to decrease the $\pi$-density of a heterocyclic system is to incorporate into the ring system a pyridine type ($\pi$-deficient) nitrogen. This would exert approximately the same electron withdrawing effect as the introduction of an electron affinic substituent. Indeed, 3-NTR exhibits approximately a 100-fold increase in sensitizing efficiency over the corresponding 4-nitroimidazole, Adams *et al.* (1979). This compound and related analogues merit further testing using *in vivo* systems to ascertain whether these structures posses the requisite pharmacological stability for use *in vivo*.

This investigation was supported by Grant No. CA 18506 to the Radiological Research Laboratory, Columbia University, awarded by the National Cancer Institute, DHHS. and The Alexander Rolston Peakcock Memorial Grant for Cancer Research from the American Cancer Society (CH 193) to the Walker Laboratory, Sloane-Kettering.

### References

ADAMS, G.E. (1982). Accomplishments, problems and prospects: A conference summary. *Int. J. Rad. Oncol. Biol. Phys.*, **8**, 805.

ADAMS, G.E., CLARKE, E.D., FLOCKHART, R. & 8 others (1979). Structure-activity relationships in the development of hypoxic sensitizers. I. Sensitization efficiency. *Int. J. Radiat. Biol.*, **35**, 133.

ASQUITH, J.C., WATTS, M.E., PATEL, K.B., SMITHEN, C.E. & ADAMS, G.E. (1974). Electron-Affinic Sensitization. V. Radiosensitization of hypoxic bacteria and mammalian cells *in vitro* by some nitroimidazoles and nitropyrazoles. *Radiat. Res.*, **60**, 108.

CHAPMAN, J.D., BLAKELY, E.A., SMITH, K.C. & URTASUN, R.C. (1977). Radiobiological characterization of the inactivating events produced in mammalian cells by helium and heavy ions. *Int. J. Radiat. Oncol. Biol. Phys.*, **3**, 97.

CHAPMAN, J.D., REUVERS, A.P. & BORSA, J. (1973). Effectiveness of Nitrofuran Derivatives in Sensitizing Hypoxic Mammalian Cells to X-rays. *Br. J. Radiol.*, **46**, 623.

COOKE, B.C., FIELDEN, E.M., JOHNSON, M & SMITHEN, C.E. (1976). Polyfunctional Radiosensitizers. I. Effects of a nitroxyl biradical on the survival of mammalian cells *in vitro*. *Radiat. Res.*, **65**, 152.

INFANTE, G.A., CAMACHO, C., PAGAN, E. & 7 others (1980). Radiosensitization studies on mouse sarcoms. In *Radiation Sensitizers, Cancer Mangement, Vol. 5*, (Ed. Brady) Masson Publ. p. 497.

RALEIGH, J.A., CHAPMAN, J.D., REUVERS, A.P., BIAGLOW, J.E., DURAND, R.E. & RAUTH, A.M. (1978). Nitropyrrole radiosensitizers: Structure function relationships. *Br. J. Cancer*, **37**, (Suppl. III) 6.

STRATFORD, I.J., ADAMS, I.J., HORSMAN, M.R. & 4 others (1980). The interaction of misonidazole with radiation, chemotherapeutic agents, or heat. In *Radiation Sensitizers Cancer Management, Vol. 5*, (Ed. Brady) Masson Publ. p. 276.

WARDMAN, P., ANDERSON, R.F., HODGKISS, R.J. & 4 others. (1982). Radiosensitization by non-nitrocompounds. *Int. J. Radiat. Oncol. Biol. Phys.*, **8**, 399.


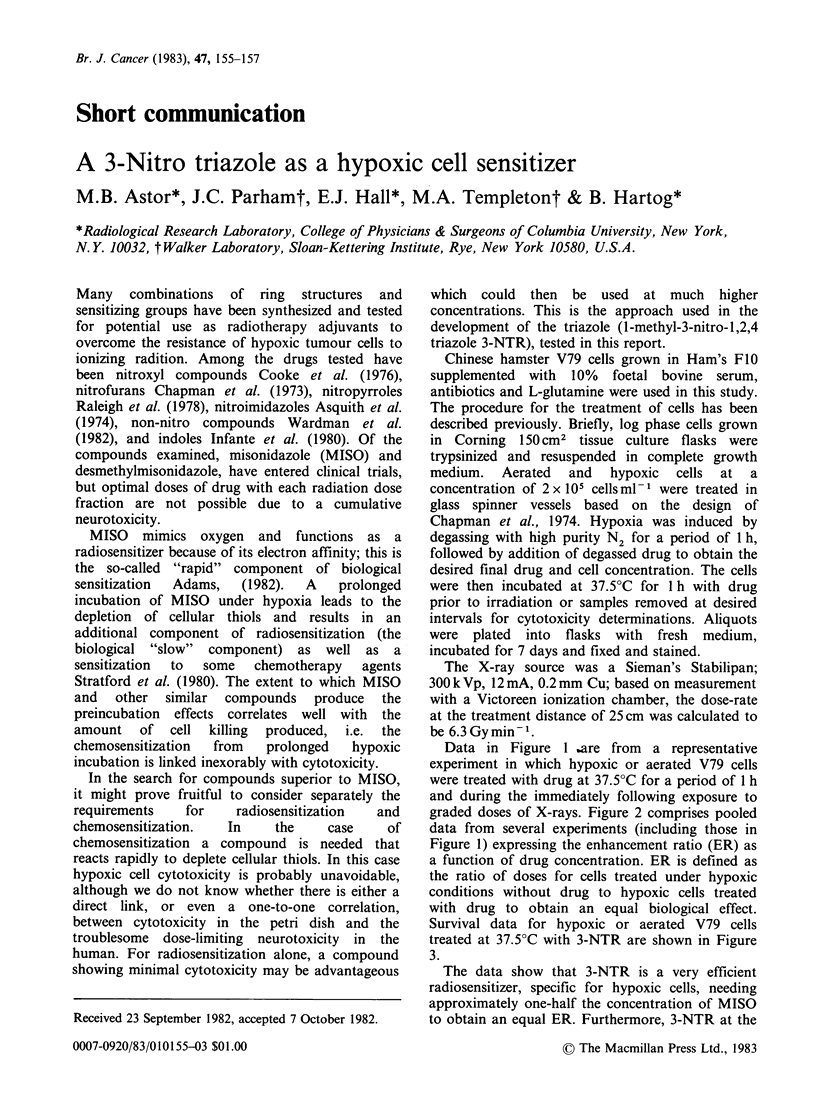

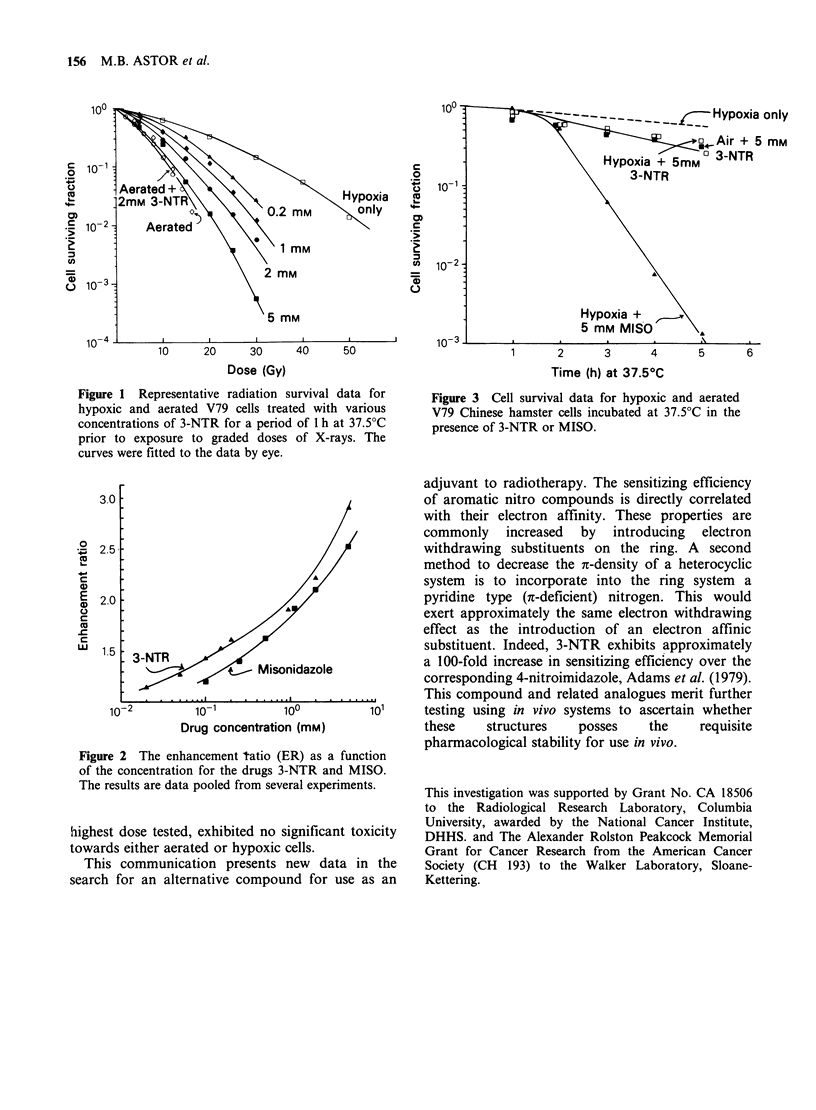

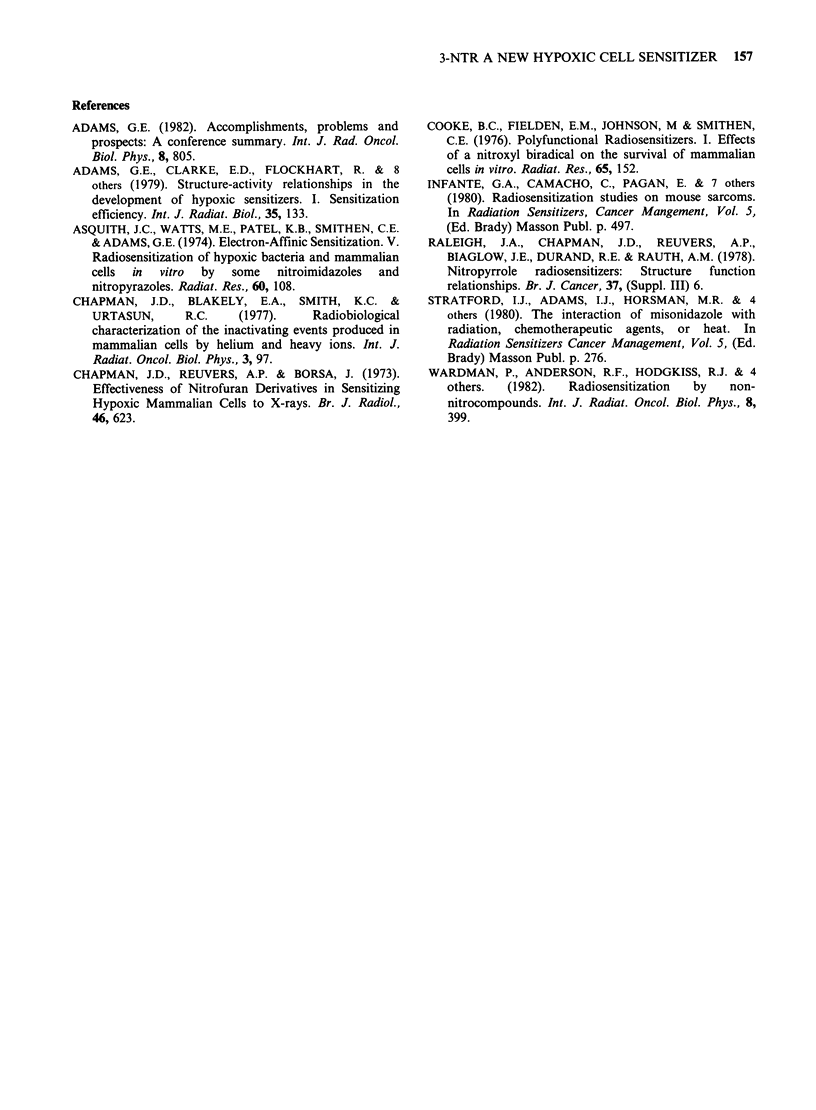


## References

[OCR_00227] Adams G. E., Clarke E. D., Flockhart I. R., Jacobs R. S., Sehmi D. S., Stratford I. J., Wardman P., Watts M. E., Parrick J., Wallace R. G. (1979). Structure-activity relationships in the development of hypoxic cell radiosensitizers. I. Sensitization efficiency.. Int J Radiat Biol Relat Stud Phys Chem Med.

[OCR_00233] Asquith J. C., Watts M. E., Patel K., Smithen C. E., Adams G. E. (1974). Electron affinic sensitization. V. Radiosensitization of hypoxic bacteria and mammalian cells in vitro by some nitroimidazoles and nitropyrazoles.. Radiat Res.

[OCR_00240] Chapman J. D., Blakely E. A., Smith K. C., Urtasun R. C. (1977). Radiobiological characterization of the inactivating events produced in mammalian cells by helium and heavy ions.. Int J Radiat Oncol Biol Phys.

[OCR_00247] Chapman J. D., Reuvers A. P., Borsa J. (1973). Effectiveness of nitrofuran derivatives in sensitizing hypoxic mammalian cells to x rays.. Br J Radiol.

[OCR_00253] Cooke B. C., Fielden E. M., Johnson M. (1976). Polyfunctional radiosensitizers. I. Effects of a nitroxyl biradical on the survival of mammalian cells in vitro.. Radiat Res.

[OCR_00278] Wardman P., Anderson R. F., Hodgkiss R. J., Parrick J., Smithen C. E., Wallace R. G., Watts M. E. (1982). Radiosensitization by non-nitro compounds.. Int J Radiat Oncol Biol Phys.

